# Resveratrol Targets a Variety of Oncogenic and Oncosuppressive Signaling for Ovarian Cancer Prevention and Treatment

**DOI:** 10.3390/antiox10111718

**Published:** 2021-10-28

**Authors:** Xue-Ling Xu, Shou-Long Deng, Zheng-Xing Lian, Kun Yu

**Affiliations:** 1 Beijing Key Laboratory for Animal Genetic Improvement, National Engineering Laboratory for Animal Breeding, Key Laboratory of Animal Genetics and Breeding of the Ministry of Agriculture, College of Animal Science and Technology, China Agricultural University, Beijing 100193, China; bs20203040330@cau.edu.cn; 2 Institute of Laboratory Animal Sciences, Chinese Academy of Medical Sciences and Comparative Medicine Center, Peking Union Medical College, Beijing 100021, China; popo84350746@163.com or

**Keywords:** resveratrol, ovarian cancer, antitumor, antioxidant

## Abstract

Ovarian cancer is a heterogeneous disease and is also the major cause of death among women from gynecologic malignancies. A combination of surgery and chemotherapy is the major therapy for ovarian cancer. Unfortunately, despite good response rates to initial surgery and chemotherapy, most patients relapse and have a generally poor survival rate. The present research sheds light on the therapeutic effects of multiple natural products in patients with ovarian cancer. Notably, these natural ingredients do not have adverse effects on healthy cells and tissues, indicating that natural products can serve as a safe alternative therapy for ovarian cancer. *Trans*-3,4,5′-Trihydroxystibene (resveratrol) is a natural product that is commonly found in the human diet and that has been shown to have anticancer effects on various human cancer cells. This review summarizes current knowledge regarding the progress of resveratrol against tumor cell proliferation, metastasis, apoptosis induction, autophagy, sensitization, and antioxidation as well as anti-inflammation. It also provides information regarding the role of resveratrol analogues in ovarian cancer. A better understanding of the role of resveratrol in ovarian cancer may provide a new array for the prevention and therapy of ovarian cancer.

## 1. Introduction

Ovarian cancer is a highly fatal disease, ranking as the 7th cancer among women worldwide. The World Ovarian Cancer Coalition Atlas 2018 showed a total of 0.239 million cases and 0.152 million deaths worldwide and predicted these numbers to rise to 0.371 million and 0.254 million, respectively by 2035.

Histopathological, ovarian cancers are classified as epithelial ovarian cancer and include serous, mucinous, endometrioid, transitional as well as clear cell carcinomas [1]. Furthermore, ovarian cancer can be classified as low-grade or high-grade tumors on the basis of genetic changes. The former are mutations in KRAS, BRAF, and PIK3CA and loss of heterozygosity (LOH) on chromosome Xq or microsatellite instability or the expression of amphiregulin; the latter has aberrations in TP53 and potential breast cancer gene 1 (BRCA1) and breast cancer gene 2 (BRCA2) aberrations as well as LOH on chromosome 7q and 9p. One of the causes of ovarian cancer may be the repeated involvement of the ovarian surface epithelium in the process of ovulation [2]. There has been substantial epidemiologic evidence demonstrating that pregnancy, breast feeding, and oral contraceptives can protect against ovarian malignancy [3]. In addition, other stimulants, such as gonadotropins or hormones, are also thought to increase the risk of ovarian cancer.

Currently, classic treatments to inhibit ovarian cancer progression is the performance cytoreductive surgery followed by a combination of platinum drugs and paclitaxel-based chemotherapy [4,5]. Eighty percent of patients respond well to treatment initially, paradoxically, though, relapse and progression to drug resistance are frequently seen [6]. Patients who receive this chemotherapy often develop cumulative toxic effects, thereby affecting their quality of life, with the 5-year survival remaining significantly low [7]. This is due to the ability of cancer stem cells (CSCs) to survive chemotherapy and restart tumor growth and metastasis [8]. For this reason, exploring other new drugs or novel treatment strategies to treat ovarian cancer may be beneficial.

Phytochemicals have been reported to modulate a mass of cellular signaling pathways with little or no toxicity to normal cells. Additionally, people are increasingly interested in using natural substances as probable chemopreventive and therapeutic agents in human populations. A vast amount of scientific evidence has demonstrated the potential of phytochemicals such as polyphenols, curcumin, and flavones in the treatment of various cancers, including ovarian cancer [9]. Resveratrol, a natural polyphenolic stilbenoid, is a phytoestrogen. Most notably, accumulative evidence supports the notion that resveratrol has immunomodulatory and anticancer properties, and its antioxidant activity and ability to inhibit enzymes may contribute to its anti-inflammatory properties.

Currently, there are several excellent articles that provide a wealth of knowledge on the chemopreventive and antitumor potential of resveratrol against various cancers [10,11,12,13]. Nevertheless, based on existing knowledge, the available literature on the utility of this natural compound for the prevention and treatment of ovarian cancer has not previously been comprehensively evaluated. Based on this background, the full potential of resveratrol in ovarian cancer prevention and treatment are discussed in the current review.

## 2. Introduction of Resveratrol

### 2.1. Sources

**Figure 1:**
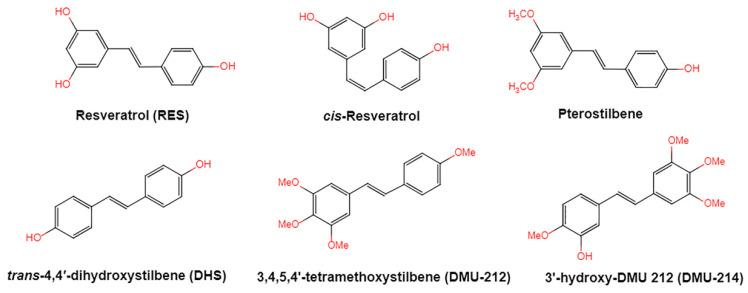
Structures of resveratrol, *cis*-Resveratrol, pterostilbene, *trans*-4,4′-dihydroxystilbene (DHS), 3,4,5,4′-tetramethoxystilbene (DMU-212), and 3′-hydroxy-3,4,5,4′-tetramethoxystilbene (DMU-214).

Resveratrol, 3,5,4′-trihydroxystilbene (Figure 1) was originally isolated in 1940 as an ingredient of white hellebore roots but was subsequently found in a variety of plants such as grapes, berries (i.e., blueberries, blackberries, cranberries and mulberries), peanuts, pines, eucalyptus, and rhubarb [14]. It is classified as a phytoalexin anti-fungicide with disease resistance in the plant kingdom. The accumulation of resveratrol in plants is due to the plant’s resistance mechanisms to parasites and other adverse conditions such as fungal infections, ultraviolet radiation, chemicals [15].

Concentrations of resveratrol vary from substance to substance. The concentration of resveratrol in blueberries is only 32 ng/g. Resveratrol concentrations in red and white wines are between 14 and 0.1 mg/L, respectively [16]. Concordantly, resveratrol concentrations in grape juice and whole grapes range from 0.05–0.5 mg/L and up to 3.54 mg/L [10]. The chemoprotective effects of resveratrol against cancer were first reported in 1997 and can be used to inhibit the tumogenesis in skin cancer [17]. Since then, resveratrol as an anticancer agent has been promoted to further research. Resveratrol has been indicated to be able to inhibit the proliferation of several types of cancer cells, including lymphoid and myeloid cancers; cancers of the breast, prostate, stomach, colon; melanoma; ovarian carcinoma; cervical carcinoma, and so on. Interestingly, researchers have discovered that high doses of resveratrol significantly extend lifespan in mammals. In addition, resveratrol can remove reactive oxygen species (ROS) and repress cyclooxygenase-1 (COX-1) or cyclooxygenase-2 (COX-2).

### 2.2. Chemistry of Resveratrol

Resveratrol is structurally based on stilbene and consists of two phenolic rings linked by styrene double bonds to form 3,4′,5-trihydroxystilbene, which exists in both the trans- and cis-iso forms. The trans-isoform is the dominant subtype and represents the most widely studied chemical form. Trans-resveratrol can undergo the *cis*-resveratrol form isomerization when exposed to solar or artificial light or ultraviolet radiation [18,19,20].

The structural modification of resveratrol has attracted the special attention of researchers, and many resveratrol derivatives, such as methoxylated, hydroxylated and halogenated derivatives, have been synthesized, showing favorable therapeutic potential [21]. Resveratrol is present in glycosylated forms in dietary products, called piceid, and maintains its biological effects and improves its overall stability and bioavailability [22]. In addition, since intestinal cells can only absorb the aglycone form of resveratrol, the absorption process requires glycosidase. Therefore, the relative content of the glycoside ligand and glycosylated resveratrol in food and beverage may regulate its absorption rate [23].

### 2.3. Absorption and Metabolism of Resveratrol

In the human intestines, 70–80% of resveratrol is rapidly absorbed by passive diffusion, while some is absorbed by forming complexes with membrane transporters such as integrins (Table 1) [24,25]. Once in the bloodstream, resveratrol can be found in three different forms: glucosidase, sulfate, or free form. In order to determine the concentration of resveratrol that can be achieved in human tissues after oral administration, patients with colorectal cancer were given 0.5–1.0 g of resveratrol once per day, and the levels of resveratrol and its metabolite, resveratrol-3-O-glucuronide, were recovered from tissues at high concentrations of 674 and 86.0 nmol/g, respectively [26]. Alternatively, circulating levels of trans-resveratrol accounted for 1.7 to 1.9% of the peak serum concentrations of total free resveratrol and conjugates in healthy males after a single oral dose of 25 mg/70 kg body weight [27].

Resveratrol was shown to interact with lipoproteins in plasma (Table 1) [32]. The plasma concentrations of trans-resveratrol on a protein basis increased with the order of their lipid content: high-density lipoproteins (HDL) < low-density lipoproteins (LDL) < very low-density lipoproteins (VLDL) [33]; in vivo, the presence of dietary polyphenolic compounds was detected in human LDL isolated from the blood samples of healthy volunteers [34,35,36]. On the other hand, the transport of glycolaldehyde and sulfate metabolites is mediated by ATP binding cassette (ABC); multidrug resistance-associated protein 3 (MRP3); ATP binding cassette, sub family C, member 3(ABCC3); breast cancer resistance protein (BCRP); and ATP-binding cassette, sub family G, member 2 (ABCG2), which are located in the basolateral and apical membranes of enterocytes. MRP3 transports resveratrol-glucuronide, and BCRP transports compounds with sulfated moiety. The absence of MRP3 or BCRP1 has pronounced effects on the disposition of resveratrol in the body [37]. Expression of BCRP and MRP3 has been detected in ovarian cancers, and these proteins have been suggested to have the ability to transport multiple anticancer agents, so these findings may be of potential clinical relevance when considering the drug treatment regimens for ovarian cancers [38,39].

Although in vitro studies have shown resveratrol to be highly potent in terms of its biologically beneficial effects in cells, its distribution in tissues is very low. This is due to resveratrol having a high metabolism, so its levels are very low. Many different metabolites are present in urine, such as resveratrol monosulfate, monosulfate dihydroresveratrol, and monoglucuronide dihydroresveratrol (Table 1) [40].

After ingestion, resveratrol travels to the intestine and then through the hepatic portal system to the liver, where it is metabolized. An initial concentration of resveratrol was detected in the blood 30 min after the oral administration of red wine to rats, and the concentration peaked after 60 min [41,42]. Tissue concentrations showed significant cardiac bioavailability and strong affinity for the liver and kidneys.

### 2.4. Bioavailability of Resveratrol

The limited bioavailability of resveratrol restricts its application and translatability. Possible methods to improve resveratrol bioavailability are developed resveratrol analogues and formulations such as adjuvants, nanoparticles, liposomes, micelles, and phospholipid complexes. In addition, several other approaches have been used to improve its bioavailability, including altering the route of resveratrol administration and blocking metabolic pathways by co-therapy with other drugs. Increasing the bioavailability of resveratrol increases its anti-tumor activity.

The exploitation of biopolymeric nanoparticles in recent years has improved the efficacy of anti-cancer drugs. The advantages of nanoparticle carriers are that they can target tumors, are more stable, and polyphenols can bind to the particles in a variety of ways, such as directly attaching themselves to the surface, becoming co-encapsulated with other compounds, or incorporating themselves within the surface of nanoparticles. As for the intraperitoneal injection of resveratrol, bovine serum albumin nanoparticles in ovarian cancer nude mice models showed higher concentrations of resveratrol in the blood and increased distribution in tissues such as liver, heart, kidney, and ovary [43]. Moreover, resveratrol–bovine serum albumin nanoparticles trigger human ovarian cancer cell line apoptosis by activating caspase [44].

Limited data in humans have demonstrated that resveratrol is quite pharmacologically safe. Currently, structural analogues of resveratrol with improved bioavailability are being sought as potential therapeutic cancer agents.

## 3. Molecular Mechanisms of Resveratrol Related to Ovarian Cancer

The influence of resveratrol on the cytochrome P450 (CYP) enzyme and cellular redox balance; the inhibition of estrogen hormone signaling; antiangiogenic and anti-inflammatory functions may all be related to its effects during the late stages of carcinogenesis.

### 3.1. Inhibition of Carcinogen Activation

Aromatase is a member of the CYP enzyme family and is encoded by the CYP19A1 gene [45]. In vivo, resveratrol blocks the activation of carcinogens via inhibiting the expression and activity of CYP1A1 that is induced by aryl hydrocarbon (Ah).

The present review sheds light on possible mechanisms by which resveratrol targets the aryl hydrocarbon receptor (AhR). It appears that this is possible by blocking the conversion of ligand-bound cytoplasmic AhR into its nuclear DNA-binding form or repressing the interaction of AhR with the transcription initiation complex on the promoter of CYP1A1 gene. Chen et al. concluded that resveratrol strongly inhibited the 2,3,7,8-tetrachlorodibenzo-p-dioxin-(TCDD)-induced AhR DNA binding activity as well as the transcription and catalytic activities of CYP1A1 and CYP1B1 in human mammary epithelial (MCF-10A) cells [46]. Furthermore, resveratrol notably reduced aromatase mRNA and protein abundance in SKBR-3 cells in a dose-dependent manner, suggesting that the compound could repress the transcriptional control dictated by promoter regulation [47].

### 3.2. Estrogen Effect

Estrogen stimulates ovarian cell proliferation and increases the metastatic potential of human ovarian cancer cell lines. The overexpression of estrogen receptor proteins has been described in more than two thirds of ovarian cancer cases [48]. Anti-estrogen therapy is one of the treatments for ovarian cancer. Understanding estrogen signaling response is essential to maximize the efficacy of anti-estrogen therapy for ovarian cancer. Studies have indicated that many antiestrogen-treated tumors maintain ER expression during relapse but that signaling through the ER pathway is altered in these resistant tumors. Therefore, targeting this pathway with resveratrol may influence the development of primary and secondary cancers.

In 1997, Gehm et al. demonstrated that resveratrol is a phytoestrogen [49]. In fact, resveratrol binds to the alpha and beta estrogen receptors (ERα and ERβ) with an affinity that is 7000 times stronger than estradiol [50]. Molecular dynamics studies have demonstrated that the binding of resveratrol to ERα is stereoselective, that is, the trans-isomer has a higher affinity for this receptor than the cis-isomer [51]. In particular, resveratrol appears to reduce estrogen signaling in the presence of ERα and ERβ, but in advanced cancer cells lacking ERβ, it is involved in tumor development.

Resveratrol can bind to estrogen receptors and can activate the transcription of antioxidant genes at concentrations that are similar to those required for its other biological effects. Resveratrol may function as a mixed estrogen agonist/antagonist in the absence of E2 but has been shown to exert antiestrogen activities in the presence of E2. Regarding its estrogenic activity, Zhang found that resveratrol-induced p53-dependent p21 gene expression and apoptosis are blocked by E2 in MCF-7 cells [52].

### 3.3. Antioxidant and Pro-Oxidant Effects

Resveratrol is an effective antioxidant that prevents the access of the oxidizing species to the lipids and scavenges free radicals before they can penetrate membrane. The antioxidant activity of resveratrol depends on the arrangement of functional groups on its nuclear structure. There is evidence indicating that the antioxidant effects of resveratrol are associated with the presence of hydroxyl groups, which are involved in the mechanisms of reducing ROS and free radicals and increasing endogenous antioxidant biosynthesis. At low doses, resveratrol interacts with the surface polar groups while localizing in the outer leaflet of the lipid bilayer at higher doses. The antioxidant activities of resveratrol are predominantly involved in the scavenging of ROS and reactive nitrogen species (RNS) and promoting the activity of a variety of antioxidant enzymes, such as superoxide dismutases (SODs), catalase, and glutathione peroxidase (GPX) [53]. Resveratrol can enhance gastrointestinal GPX promoter activity in HepG2 cells [54]. The antioxidant properties of resveratrol can also be attributed to its ability to decrease copper-mediated oxidation and the prevention of LDL and cell membranes lipid peroxidation [55,56,57].

It has been reported that the antioxidant properties of resveratrol have been successfully used to protect cells against hydrogen peroxide induced by oxidative stress and that pretreatment with resveratrol promotes cell survival and prevents cell death induced by ultraviolet radiation. Additionally, resveratrol increased SOD and GPX activity and retarded malondialdehyde levels in senescence-accelerated mice models at different doses given over 8 weeks [58].

In practice, every antioxidant is a redox agent, and resveratrol is no exception. It also undergoes an autooxidation process, leading to the production of H_2_O_2_ and complex mixtures of semiquinones and quinones. Copper is the most redox-active metal that exists in the nucleus, serum, and tissues [59]. Approximately 20% of copper is stored in the nucleus and binds with DNA both at intrastrand and interstrand levels [60]. Resveratrol possesses pro-oxidation properties that lead to the oxidative breakage of cellular DNA in the presence of transition metal ions such as copper [61]. In recent years, research has suggested that the pro-oxidative potential may be a shared mechanism for the anticancer and chemoprophylaxis characteristics of polyphenols. Copper ions are reported to be increased in various malignancies, so the present study might explain the anticancer activity of resveratrol in various cancer cell lines [62].

### 3.4. Inhibition of Angiogenesis

Angiogenesis is an important agent of tumor development. Sustained expansion of a tumor mass requires new blood vessel formation to provide rapidly multiplying tumor cells with sufficient oxygen and metabolites. However, because rapidly proliferating cells increase metabolic activity and oxygen consumption, tumors may maintain an intratumoral hypoxic environment [63]. The key regulator of hypoxia-induced angiogenesis is the transcription factor hypoxia inducible factor (HIF-1). Vascular endothelial growth factor (VEGF), basic fibroblast growth factor (bFGF), VEGF receptor (VEGFR), IL-8, inducible nitric oxide synthase (iNOS), and angiopoietin have been identified as HIF-regulated angiogenic factors [64].

A large number of studies have shown that resveratrol has anti-angiogenic effects. Resveratrol substantially induced HIF-1α protein degradation through the proteasome pathway and also greatly inhibited VEGF expression and thus provided a novel potential mechanism for inhibiting human ovarian cancer progression [65]. Furthermore, Garvin et al. observed notably lower tumor growth, significant increases in apoptosis, and decreased angiogenesis in ERα- ERβ+ MDA-MB-231 breast tumors in resveratrol-treated nude mice [66].

### 3.5. Anti-Inflammatory Effects of Resveratrol

Inflammation is a major driver of carcinogenesis, acting at all stages of tumorigenesis [67,68]. Components of inflammatory pathways, including free radicals, cytokines, nuclear transcription factor-kappa B (NF-κB), signal transduction and transcriptional activator 3 (STAT3), iNOS, COX-2, prostaglandin, and VEGF, have been proven to be associated with the development of numerous malignant tumors, including ovarian cancer. In ovarian cancer samples, COX-2 was found to be highly expressed in non-mucinous ovarian cancer, and COX-2 expression was significantly associated with adverse prognostic factors, such as stage, tumor grade, residual disease status, and the presence of ascites [69].

Previous hypotheses regarding the causes of ovarian cancer have attributed risk to an excess number of lifetime ovulations or to elevations in steroid hormones. Additionally, inflammation may underlie ovulatory events because an inflammatory reaction is induced during the process of ovulation [70,71]. Components of the inflammatory pathway include STAT3, NF-κB, iNOS, COX-2, and VEGF [72]. In addition, in a population-based, case–control study, the long-term use of nonsteroidal anti-inflammatory drugs (NSAID) was negatively associated with ovarian cancer risk [73].

Phytochemicals play an anticancer role by regulating various signaling pathways, one of which is inflammatory signaling. The inhibitory effects on tumor growth that are provided by resveratrol are, in part, mediated through its anti-inflammatory activity. A lot of their molecular targets occur on iNOS, COXs, leukotrienes, NF-κB, tumor necrosis factor-alpha (TNF-α), interleukins (ILs), etc. [74]. In murine and human macrophage cells, resveratrol blocks the TNF-induced activation of NF-κB in a dose- and time-dependent manner [75,76]. Resveratrol also inhibits reactive oxygen intermediate generation and lipid peroxidation induced by TNF.

Many other inflammation-related proteins, such as AKT (i.e., protein kinase B), lysophosphatidic acid (LPA), and protein kinase C (PKC) are also overexpressed in more than 70% of ovarian cancers [77]. Interestingly, LPA has also been found to cause the upregulation of IL-6, IL-8, and VEGF in epithelial ovarian cancer (EOC) cell lines through the Gi/phosphoinositide 3-kinase (PI3K)-AKT/NF-κB pathway [78]. In ovarian cancer cells, resveratrol inhibits the expression of HIF-1α. The underlying mechanism of inhibition appears to be responsible for both the inactivation of mitogen-activated protein kinase (MAPK) and p70S6K, resulting in a profound decrease in VEGF expression and cell migration [79]. Collectively, these data suggest that resveratrol may inhibit ovarian carcinogenesis by downregulating NF-κB activity and blocking HIF-1α and VEGF expression. The regulation of mal-regulated inflammatory pathways is a promising chemoprophylaxis strategy against ovarian cancer.

## 4. Effect of Resveratrol on Prevention and Treatment of Ovarian Cancer

### 4.1. In Vitro

Resveratrol has been proven to enhance apoptosis in human ovarian cancer cell lines, leading to compromised cell proliferation [80] (Table 2).

#### 4.1.1. Anti-Proliferative and Apoptosis Inducing Activity

**Figure 2:**
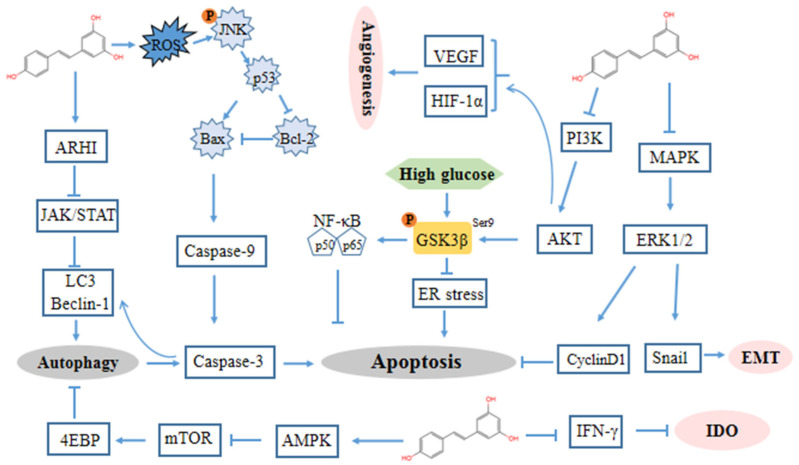
Mechanism of resveratrol against ovarian cancer. Extracellular signal-regulated kinase (ERK) 1/2, aplasia ras homologue member I (ARHI), AMP-activated protein kinase (AMPK), interferon-γ (IFN-γ), Indoleamine 2, 3-dioxygenase (IDO), epithelial-to-mesenchymal transition (EMT).

The majority of evidence implies that the antiproliferative and growth inhibitory effects of resveratrol are due to its ability to block DNA synthesis and interfere with various stages of cell cycle progression. Tumor cells increase glucose uptake and then generate a large number of metabolites through aerobic glycolysis to satisfy their rapid and unlimited growth [96]. Glycogen synthase kinase-3β (GSK3β) was identified as a kinase that phosphorylated and inactivated glycogen synthase, thereby relatively increasing glucose metabolism [97,98]. In ovarian cancer cells, resveratrol induces endoplasmic reticulum (ER) stress-mediated apoptosis by suppressing the hexosamine biosynthesis pathway and disrupting protein glycosylation via activated GSK3β by suppressing the inhibitory S9-phosphorylation of GSK3β (Figure 2) [83].

In particular, the effects of resveratrol on ovarian cancer cells (OVCAR3) were investigated using a proteomic method, which implied that resveratrol downregulated the phosphorylation of AKT and GSK-3β at Ser9 in a concentration dependent manner and reduced phosphorylated extracellular signal-regulated kinase (ERK) 1/2 in ovarian cancer cells, thereby inhibiting cyclin D1 expression [84].

In addition, VEGF and HIF-1α are elevated in several human tumors and their metastases and are strongly associated with more aggressive tumor phenotypes [99]. In a study, resveratrol treatment inhibited the expression of VEGF and HIF-1α in A2780/CP70 and OVCAR-3 cells by reducing PI3K/AKT and MAPK activation, thereby inhibiting the progression and angiogenesis of human ovarian cancer [65].

A great deal of evidence has also highlighted the role of STAT3 signaling in promoting carcinogenesis and tumor progression by upregulating gene expression and promoting dysregulated growth, survival, and angiogenesis as well as modulating immune responses [100,101]. Compounds that inhibit JAK/STAT signaling have been reported to inhibit the growth of ovarian cancer cells [102].

Zhong et al. used two human ovarian cancer cell lines, OVCAR-3 and CAOV-3, to study the inhibitory roles of resveratrol on ovarian cancer cells [85]. The results demonstrated that human ovarian cancer cells treated with resveratrol had a remarkable accumulation of the G1 phase and increased apoptotic fraction. Furthermore, they concluded that this was associated with the inhibition of STAT3 signaling and its downstream cancer-related gene expression. Given the function of resveratrol in ovarian cancer cells, it could be considered that resveratrol may be a promising candidate in the management of ovarian cancers, especially the ones with cis-platinum resistance.

#### 4.1.2. Modulate Autophagy

Autophagy serves a dual role in ovarian cancer, and the modulation of autophagy contributes to the inhibition of ovarian cancer development. However, increased autophagy is also related to chemotherapy resistance. In one study, the analysis of five resveratrol-treated human ovarian cancer cell lines (i.e., A2780, CaOV3, ES-2, TOV112D, and A1947) by light and electron microscopy revealed morphology and ultrastructural changes that suggested that autophagy was the dominant mode of cell death (with apoptotic death also present) [86].

There is ample evidence that autophagy is dependent on ATG5/ATG7 and that it is associated with microtubule-associated protein light chain 3 (LC3) truncation and lipidation. Lang et al. reported that the hallmarks of autophagy, LC3 and ATG5, were both upregulated in resveratrol-treated cancer cells (OVCAR-3 and Caov-3) [87]. The results demonstrated that resveratrol induces the production of ROS, triggering autophagy and subsequent apoptotic cell death. The inhibition of autophagy significantly reduced resveratrol-induced cell death and caspase 3 activity in human ovarian cancer cells.

Beclin 1 has a central role in autophagy and interacts with several cofactors to regulate the formation and maturation of autophagosomes [103]. Zhong et al. suggested that resveratrol inhibited STAT3, upregulated LC3 and Beclin-1 and enhanced the autophagy activity of ovarian cancer cells, resulting in the death of cancer cells [88]. One of the tumor suppressive effects of aplasia ras homologue member I (ARHI) is to attenuate STAT3 signaling. Alternatively, resveratrol promotes ARHI re-expression and prevents STAT3 translocation to the nucleus and localization in focal adhesion complexes, then initiates autophagy and induces tumor dormancy [104]. ARHI was upregulated after resveratrol treatment accompanied by decreased STAT3 expression and p-STAT3 generation. ARHI-specific siRNA transfection increased the formation of RanGTPase-importin β complex in resveratrol-treated CAOV-3 and OVCAR-3 cells, enhanced STAT3 nuclear translocation, and increased the total cell number to a limited extent [89]. Concordantly, Ferraresi provided evidence that resveratrol increased autophagy via the promotion of Beclin-1 and LC3 through the upregulation of ARHI and the inactivation of STAT3, thereby counterbalancing the IL-6 induction of ovarian cancer cell migration [90]. Furthermore, they demonstrated that resveratrol inhibits mTOR complex 1 by repressing AKT and activating AMP-activated protein kinase (AMPK), inducing autophagy that drives cancer cells into a non-replicating, dormant state [91].

#### 4.1.3. Attenuation of Immune-Suppressive Microenvironment via Inhibition of IDO

Indoleamine 2, 3-dioxygenase (IDO) is a heme-containing enzyme that catalyzes the initial rate-limiting step in tryptophan degradation. IDO has been shown to be a crucial mediator of tumor-mediated immune tolerance, contributing to immune evasion [105]. Synergistic IDO expression in response to IFN-gamma and TNF-alpha requires increases in NF-kappaB translocation [106]. The elevation of IDO generates an immunosuppressive microenvironment by inhibiting T lymphocyte function and potently activating regulatory T cells (Tregs) in lymph nodes [107]. Resveratrol can inhibit the expression and activity of interferon-γ (IFN-γ)-induced IDO [108] (Figure 2).

#### 4.1.4. Sensitization Chemo-Sensitizing Effects

The predominant obstacle for the treatment of advanced ovarian cancer is resistance to chemotherapeutic drugs. The multidrug resistance protein 1 (MDR-1) mediated the increased drug efflux pump activity, increased DNA damage repair ability, reduced drug intake, and reduced apoptosis and drug-induced cell cycle arrest that are responsible for chemotherapy resistance in ovarian cancer [109]. Abnormal activation of NF-κB enhances drug resistance and protects cancer cells against apoptosis induced by pharmacological drugs, so most cancer preventive agents act as NF-kB inhibitors [110]. Nessa et al. concluded that the administration of resveratrol 2 h before platinum drugs sensitized ovarian cancer cells to platinum-induced apoptosis via the downregulation NF-κB, thus increasing the efficacy of the platinum drug [82]. Engelke et al. found that resveratrol could improve the efficacy of cisplatin in ovarian cancer by modulating molecular targets, including the EGFR or VEGFR family of receptor tyrosine kinases [92].

#### 4.1.5. Inhibition of Epithelial Mesenchymal Transition and Metastasis

The danger of ovarian cancer is that cancer cells can undergo rapid and early metastasis to the peritoneum and the omentum as well as to organs located in the peritoneal cavity [111]. A pervious study provided evidence that resveratrol inhibits the adhesion of ovarian cancer cells to human peritoneal mesothelial cells (HPMCs) by decreasing cellular α5β1 integrin levels and by increasing hyaluronic acid secretion to the extracellular matrix (ECM) [93].

LPA, a bioactive phospholipid, is a potent inducer of tumor cell migration, proliferation, survival, and angiogenesis [112,113]. The high expression of VEGF reflects ovarian carcinoma spread and poor prognosis [114]. Resveratrol blocked the expression of HIF-1α and VEGF, effectively inhibiting LPA-induced migration of human ovarian cancer cells under hypoxia conditions [79].

Matrix metalloproteinases (MMPs) are a family of strongly related enzymes that degrade the ECM. Ovarian tumor cells and the surrounding stromal cells stimulate the synthesis of various MMPs, leading to the increased invasion and metastatic potential of cancer cells [115]. MMP-1 co-locates with other MMPs and proteases in malignant effusion cells, primary tumors, and metastatic lesions of ovarian cancer [116,117,118]. The MMP-1-protease activated receptor 1 (PAR1) signaling axis is involved in EOC invasion, and siRNA silencing MMP-1 and PAR1 both significantly reduced LPA-induced EOC invasion [119]. Polymorphisms in the MMP promoter may generate the overexpression of MMPs in ovarian cancer. An earlier study provided a correlation between MMP-1 gene expression and the insertion or deletion of guanine nucleotides in the MMP-1 promoter region in ovarian cancer [120]. A study also reported that ovarian cancer patients with a common G/GG MMP-1 promoter polymorphism have shortened survival [121].

A study showed that treatment with resveratrol on A2780 and A2780CP ovarian cancer cells increased cell death in a dose-dependent manner and inhibited cisplatin-induced the epithelial-to-mesenchymal transition (EMT) [94]. This is because resveratrol inhibits cisplatin-induced snail expression by reducing ERK pathway activation, reversing the morphological changes induced by cisplatin and reducing cell migration.

#### 4.1.6. Impaired Ovarian CSCs

In recent years, natural products have been considered to interfere with the CSC self-renewal process [122]. It has been demonstrated that CSCs can be found in the ascites derived from ovarian cancer patients and mice inoculated with human ovarian cancer cell lines, which are also identified by flow cytometry and Hoechst 33342 dye efflux analysis as side population (SP) cells [123].

CSCs are responsible for one of the poor prognoses of ovarian cancer. Seino and colleagues investigated the effects of resveratrol on the viability and self-renewal capacity of CSCs derived from A2780 human ovarian cancer cells [95]. They also provided evidence that at certain concentrations, resveratrol effectively kills ovarian CSC independently of ROS, while ROS dependently reduces the self-renewal capacity of the ovarian CSCs that survived resveratrol treatment.

### 4.2. In Xenograft Models

A large number of trials have begun to determine whether resveratrol is active in xenograft models of ovarian cancer in mice to assess the extent to which the compound selectively targets tumor cells relative to normal tissues (Table 3).

In a mouse model of ovarian cancer, resveratrol can reduce tumor progression due to increased cytotoxic T lymphocyte (CTL) and antigen-presenting cells in tumor tissues. A remarkable decrease in transforming growth factor-β (TGF-β) and increased secretion of IFN-γ were further observed [80]. In another mouse model, resveratrol-treated mice showed a marked decrease in tumor uptake of glucose and attenuated ovarian tumor growth [81].

Resveratrol reduced the average volume and mass of PA-1 cell xenografts in athymic nude mice and retarded the expression of proliferating cell nuclear antigen (PCNA) and eukaryotic elongation factor 1A2 (eEF1A2), as well as activated caspase-3 [124]. In addition, the attenuation of CD31 positivity and microvessel density in PA-1 cell xenografts also implicates the antiangiogenic effect of resveratrol in ovarian cancer.

Another study reported that resveratrol diminished STAT3 expression, decreased p-STAT3 nuclear translocation and ARHI upregulation to inhibit orthotopic tumor growth in terms of significantly reduced tumor sizes, ascitic volume, ascitic cancer cell numbers, and CA125 levels, while maintaining reproductive fertility in female rats [125].

## 5. Effects of Resveratrol in Combination with Other Compounds on Ovarian Cancer

The natural compound resveratrol may have a single anti-cancer effect. Accumulating evidence suggests as a potential anticancer therapy, synergistic combinations of resveratrol with different compounds may further enhance its anticancer efficacy and scope of action (Table 4).

The combination of resveratrol and its derivative acetyl resveratrol effectively reduces cell proliferation and metabolism in ovarian cancer cell line SKOV-3 aggregates. Additionally, growth restriction is associated with the reduced VEGF secretion that is controlled by NF-κB protein [126].

Combined treatment with indole-3 Carbinol (I3C) and resveratrol resulted in G0/G1 cell cycle arrest and increased apoptosis in SKOV-3 ovarian cancer cells through the upregulation of tumor suppressor protein P21 and the inhibition of the retinoblastoma protein (Rb) and anti-apoptotic protein survivin (SVV) expression [127].

Polyphenols, such as resveratrol, quercetin, catechin, and epicatechin, have been proven to be effective in inducing S-phase cell cycle arrest through the bimodal modulation of nitric oxide (NO)/NOS in T47D cells.

Resveratrol along with the combination treatments of nutlin-3 and TGF-β induced cytochrome c release, leading to caspase-3 activation, and effectively induced the apoptosis of the human ovarian cancer cell line A2780/CP70 [128]. During this combination, resveratrol at a low concentration (5 μM) was able to achieve the same apoptotic potential as monotherapy with a high concentration (25 μM).

In combination with chemotherapy drugs, resveratrol can enhance and reduce cancer cell death. The combination of a new monofunctional planaramineplatinum(II) complex, namely tris (8-hydroxyquinoline) monochloroplatinum(II) chloride (coded as LH3) and resveratrol may be beneficial in stimulating NF-κB, thereby reducing cell death [129]. This phenomenon may be due to the downstream processes that are responsible for cell death, interfering with each other to produce antagonistic outcomes.

Adriamycin, a potent antineoplastic agent, has a very wide antitumor spectrum that can be used in the treatment of ovarian cancer. Fatease and colleagues developed a polyphenol micellar system that evaluates the efficacy of ADR-sensitive (ES2-LUC) and ADR-resistant (A2780ADR) ovarian cancer cells when ADR was administered in combination with micellar resveratrol: quercetin or micellar resveratrol: curcumin [130]. The results suggest that in a A2780ADR xenograft mouse model, only the ADR+ micellar resveratrol: quercetin treatment group showed significantly reduced tumor growth, whereas in the ES2-LUC xenograft mouse model, all treatment groups had pronounced effects on tumor volume.

## 6. Analogs of Resveratrol against Ovarian Cancer

The low bioavailability of resveratrol limits its therapeutic applications. Numerous studies have shown that resveratrol analogues have stronger anti-proliferation ability.

Pterostilbene, a resveratrol analog with improved bioavailability, has been shown to offer antioxidant and anticancer properties. Pterostilbene was observed to cause apoptosis and to inhibit cell cycle progression in both OVCAR-8 and Caov-3 cells by downregulating anti-apoptotic proteins, such as MCL-1 and BCL-2, and cell cycle proteins, such as cyclin D1 [131]. Pterostilbene has also been reported to activate caspase 3 and 9 and to induce the ROS-mediated intrinsic pathway for the apoptosis of SKOV-3 cells [132]. Moreover, pterostilbene decreased the release of TNF-α cytokines in IGROV-1 cells by inhibiting the NF-κB, AKT, and ERK pathways and helped to reduce the effect activity and translocation of NF-κB in the treatment of ovarian cancer [133].

Another analogue, *trans*-4,4′-dihydroxystilbene (DHS) (Figure 1), can reduce cell ribonucleotide reductase (RNR) activity, resulting in reduced deoxyribonucleoside triphosphates (dNTPs) synthesis, DNA damage, the arrest of cells at S-phase, and ultimately, apoptosis. Moreover, the combination of DHS and cisplatin was efficacious in inhibiting tumor growth, and resistance to cisplatin in ovarian cancer could be overcome by DHS [134].

The compound 3,4,5,4′-tetramethoxystilbene (DMU-212) (Figure 1) arrested cell cycle development in the G2/M or G0/G1 phase, which caused the apoptosis of two ovarian cancer cell lines, A-2780 and SKOV-3 [135]. CYP1B1 and CYP1A1 are involved in the carcinogenic process, in which CYP1B1 mRNA and protein overexpression have been detected in a variety of malignant tumors. The mRNA and protein expression of CYP1A1 and CYP1B1 were decreased in A-2780 and SKOV-3 cells treated with DMU-212. It is noteworthy that DMU-212 resulted in the entire inhibition of CYP1B1 protein expression in A-2780 cells.

Compared to those in SKOV-3 cells, the metabolites 3′-hydroxy-3,4,5,4′-tetramethoxystilbene (DMU-214) (Figure 1) of resveratrol analogue 3,4,5,4′-tetramethoxystilbene (DMU-212) demonstrated better anti-proliferation and pro-apoptotic actions of DMU-214 in the A-2780 cell line [136]. DMU-214 downregulated cyclin B1 mRNA levels, leading to p53-dependent cell apoptosis. In light of these findings, they evaluated the antitumor potential of DMU-214 in human ovarian cancer xenografts in mice. Severely compromised immunodeficient (SCID) mice injected with A-2780 cells were found to have significantly reduced tumor growth in mice treated with DMU-214 compared to untreated controls. DMU-214 also downregulated the mRNA and protein levels of serum response factor (SRF) in the SKOV-3 cells, which effectively impaired tumor dissemination [137]. At the same time, the results of microarray analysis also revealed its negative effects on the proliferation-related genes of SKOV-3 cells.

Vergara and colleagues designed new analogues of resveratrol in which the C-C double bond of the natural derivative was replaced by diaryl-substituted imidazole analogues [138]. They further evaluated the anti-tumorigenic activity of these compounds in ovarian cancer cell line SKOV-3 and in primary cancer cells isolated from the ascites of patients with metastatic ovarian cancer. Compared to resveratrol, the result revealed that the resveratrol analogues exhibited enhanced anti-proliferative ability. The analogue-induced antiproliferative effects were via the inhibition of AKT, GSK, and ERK phosphorylation the decrease of the protein expression of cyclin D1, Bcl2, and β-catenin. Furthermore, it also can reduce cell adhesion to ECM components.

## 7. Conclusions and Future Directions

An ideal chemo-preventive agent could modulate normal growth control to a preneoplastic or cancerous cell population by modifying aberrant signaling pathways or by inducing apoptosis in cells that could not be repaired. It should target the multiple biochemical and physiological pathways that support tumor development while having little or no toxicity against healthy tissues.

Polyphenols have a number of properties, including antioxidant and anti-inflammatory activities [139]. They probably modulate their anti-inflammatory effects by DNA methylation and histone modification. And of all the anticancer polyphenols that have been studied, resveratrol possesses the widest evidence supporting its direct and indirect anticancer properties [140]. In animal models, resveratrol has minimal toxicity, and even the actively proliferating tissues such as bone, marrow, and the gastrointestinal tract are not adversely affected. In fact, the minimal toxicity of resveratrol in animals has helped it become used in human studies sponsored by the National Cancer Institute that are aimed at cancer prevention in healthy volunteers. Taken together, these observations suggest that resveratrol can be used as a useful complementary medicine adjunct for the prevention and treatment of ovarian cancer due to its natural source, safety, and low cost compared to cancer drugs.

Nevertheless, a practical question with resveratrol is whether the same levels of growth inhibition seen in serum or tissue concentrations can be achieved in vitro. The pharmacokinetics and bioactivity of resveratrol and its metabolites are not understood in humans well enough to determine this. Further research is needed on this natural compound. The following issues require greater attention in future studies: (i) randomized clinical trials should be conducted to define the therapeutic efficacy of resveratrol in ovarian cancer; (ii) the effects of resveratrol combined with conventional chemotherapy, target therapy or immunotherapy remain to be further investigated; and (iii) new, more potent compounds that mimic the effects of resveratrol should be developed.

## Figures and Tables

**Table 1:** Human oral administration of resveratrol metabolites in different liquids and tissues.

Structure	Metabolite	Location	Reference
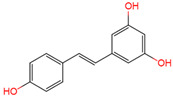	*trans*-resveratrol	serum, plasma, urine	[24,28,29]
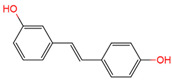	3,4′-O-dihydroxy-trans-stilbenes	urine	[29]
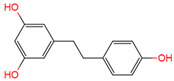	dihydroresveratrol	urine, plasma	[29,30]
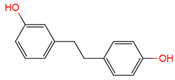	lunularin	urine	[29]
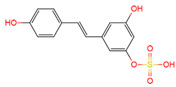	*trans*-resveratrol-3-O-sulfate	plasma, urine	[24,31]
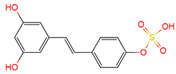	*trans*-resveratrol-4′-O-sulfate	plasma, urine	[24,31]
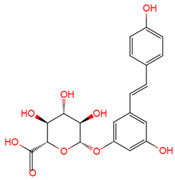	*trans*-resveratrol-3-O-glucuronide	serum, plasma, urine	[24,28,31]
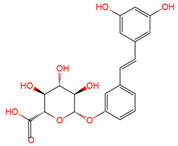	*trans*-resveratrol-4′-O-glucuronide	serum, plasma, urine	[28,31]
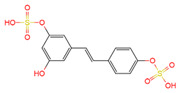	*trans*-resveratrol-3,4′-O-disulfate	plasma	[31]

**Table 2:** The antitumor effect and mechanisms of action of resveratrol in ovarian cancer.

Cell Lines	Dose & Time	Anticancer Effects	Mechanisms	Reference
A2780, SKOV3	25, 50 µM; 72 h	Inhibited the regrowth of A2780 cells but had no effect on SKOV3 cells	Inhibited glycolytic response	[81]
A2780, A2780^cisR^ and A2780^ZD0473R^	Variable; 24 h, 72 h	Decreased the resistance to the platinum drugs	↓NF-κB	[82]
PA-1, MDAH2774 and SKOV3	50 µM; 24 h, 48 h	Inhibited cell growth	↑Apoptosis; ↑ER stress; ↓protein glycosylation;↓p-AKT; ↓GSK3β (phosphorylated at S9)	[83]
OVCAR-3, SKOV3	>50 µM; 30 min, 24 h, 48 h and 72 h	Inhibited proliferation, decreased drug resistance	↓cyclin D1; ↓p-AKT; ↓p-GSK3β; ↓p-ERK	[84]
A2780/CP70, OVCAR-3	12.5, 25, 37.5, 50, 75, 100, 150 μm; 6 h, 12 h	Inhibited ovarian cancer progression and angiogenesis	↓HIF-1α protein;↓VEGF;↓AKT and MAPK phosphorylation	[65]
OVCAR-3	120 μM; 48 h	Caused growth arrest and apoptosis	↓HES1; ↓ Notch2; ↑E-cadherin; ↓p-STAT3; ↓SVV; ↓c-Myc; ↓Bcl-2	[85]
CAOV-3	120 μM; 48 h	Increased apoptosis fraction; G1 phase accumulation	↓HES1; ↓Notch2;↓Wnt2; ↓p-STAT3;↓SVV; ↓c-Myc; ↓Bcl-2	[85]
A2780, CaOV3, ES-2, TOV112D, A1947	50–200 μM; Variable	Inhibits the proliferation and survival of ovarian carcinoma cells	↑Cytochrome c;↑Caspase 9	[86]
OVCAR-3 and Caov-3	100 µM; 48 h	Inhibited cell growth, triggered autophagy	↑Apoptosis; ↑ROS generation; ↑ATG5; ↑caspase 3; ↑cleavage from LC3-I to LC3-II	[87]
OVCAR-3 and Caov-3	100 µM; 48 h	Distinct growth arrest, increased autophagy	↑Apoptosis; ↓STAT3; ↓p-STAT3; ↑Beclin-1;↑cleavage from LC3-I to LC3-II	[88]
OC-CAOV-3 and OVCAR-3	100 µM; 24 h, 48 h, 72 h	Reduced proliferation activities, increased autophagy	↑Apoptosis; ↑ARHI;↓p-STAT3; ↓the metastasis induced by IL-6	[89]
NIH-OVCAR3	100 µM; 24 h, 48 h, 72 h	Inhibited cell migration	↑ARHI; ↓p-STAT3;↑Beclin-1; ↓p-AKT;	[90]
NIH-OVCAR3	100 µM; 1 h, 4 h, 24 h	Inhibited protein synthesis and cell growth, induced autophagy.	↓mTORC1;↓p-AKT; ↑p-AMPK;↓p-4EBP1; ↑p-eIF2α	[91]
A2780	10 µM; 48 h, 72 h, 26 weeks	Decreased proliferation, inhibited cell migration, prevented the development of cisplatin resistance	↑ROS; ↓p-EGFR; ↓p-ErbB2; ↓p-ErbB3	[92]
A2780 and SKOV3	Variable; 72 h	Inhibited cell adhesion, decreased metastasis.	↓α5β1 integrin; ↑HA	[93]
OVCAR-3 and CAOV-3	Variable; 2 h	Inhibited LPA and hypoxia-induced cell migration	↓HIF-1α;↓VEGF; ↓p-ERK; ↓p70S6Kinase	[79]
A2780 and A2780CP	0–60 µM; 24 h, 48 h, 72 h	Increased cell death; inhibited cell migration	↓p-ERK; ↓Snail; ↑LC3B-II	[94]
A2780 CSCs	Variable; 24 h, 48 h	Inhibits the survival of cells; increases the intracellular ROS level	↑ROS; ↓Sox2; ↓Nanog	[95]

↓: downregulated; ↑: upregulated; NF-κB, nuclear transcription factor-kappa B; ER, endoplasmic reticulum; AKT, protein kinase B; GSK3β, glycogen synthase kinase-3β; HIF-1α, hypoxia-inducible factor-1α; VEGF, vascular endothelial growth factor; MAPK, mitogen-activated protein kinase; HES1, split homolog-1; SVV, survivin; p-STAT3, phosphorylated signal transducer and activator of transcription 3; OC, ovarian cancer; ROS, reactive oxygen species; ATG5, autophagy related 5; LC3, microtubule-associated protein light chain 3; ARHI, aplasia ras homologue member I; IL, interleukin; mTORC1, mammalian target of rapamycin complex 1; AMPK, AMP-activated protein kinase; p-4EBP1, phosphorylated 4E-binding protein 1; eIF2α, eukaryotic initiation factor 2; p-EGFR, phosphorylated epidermal growth factor receptor; α5β1, alpha5beta1; HA, hyaluronic acid; p-ERK, phosphorylated extracellular signal-regulating kinase; SOX2, SRY-box transcription factor 2; CSCs, cancer stem cells.

**Table 3:** Effect of resveratrol in xenograft models of ovarian cancer.

Animal Models	Dose and Route	Anticancer Effects	Mechanisms	Frequency and Duration	Reference
C57BL/C mice were subcutaneously inoculated with ID8 cells	Amount of 50 or 100 mg/kg body weight; IP	Dramatically decreased tumor weight	↓TGF-β; ↑IFN-γ	Daily; 21 days	[80]
A fluorescent xenograft mouse model of ovarian cancer	Amount of 160 mg/kg; IP	Decreased tumor volume	Decreased the uptake of glucose	Daily; 14 days	[81]
Female BALB/c mice injected with PA-1 cells	Amount of 50 or 100 mg/kg body weight; IP	Inhibited the tumor cell proliferation	↓PCNA; ↓CD31; ↓eEF1A2; ↑caspase-3	Daily; 4 weeks	[124]
A rat orthotopic OC model was established using the rat NUTU-19 OC cell line	Amount of 20 mg/kg/day; IP	Effectively inhibited rat orthotopic ovarian cancer growth without affecting normal tissues	↓STAT3; ↓p-STAT3; ↓serum CA125 levels; ↑ARHI; ↑PIAS3	Daily; 2 weeks	[125]

↓: downregulated; ↑: upregulated; injected intraperitoneally (IP); TGF-β, transforming growth factor-beta; IFN-γ, interferon-gamma; PCNA, proliferating cell nuclear antigen; CD31, platelet endothelial cell adhesion molecule-1; eEF1A2, eukaryotic elongation factor 1A2; STAT3, signal transducer and activator of transcription 3; p-STAT3, phosphorylated signal transducer and activator of transcription 3; CA125, carbohydrate antigen 125; ARHI, age-related hearing impairment; PIAS3, protein Inhibitors of Activated STAT3; OC, ovarian cancer.

**Table 4:** Effects of resveratrol in combination with other compounds.

Combination	Cell Lines	Effect	Mechanisms	Reference
Resveratrol + acetyl-resveratrol	SKOV-3	Inhibited cell growth	↓VEGF; ↓NF-κB; ↑IL-8	[126]
Resveratrol + I3C	SKOV-3	Resulted in cell cycle arrest; increased apoptosis	↑tumor suppressor protein p21; ↓Rb; ↓SVV	[127]
Resveratrol + nutlin-3 +TGF-β	A2780/CP70	Induced apoptosis	↑caspase-3; ↑caspase-9; ↑cytochrome c	[128]
Resveratrol + LH3	A2780, A2780 ^cisR^ and A2780 ^ZD0473R^	Reduced cell death	↑NF-κB	[129]
Micellar resveratrol + quercetin + ADR	A2780ADR xenograft model	Reduced tumor growth	Chemosensitization	[130]
Micellar resveratrol + quercetin + ADR; micellar resveratrol + curcumin + ADR	ES2-Luc xenograft model	Reduced tumor volume	Chemosensitization	[130]

↓: downregulated; ↑: upregulated; VEGF, vascular endothelial growth factor; NF-κB, nuclear transcription factor-kappa B; I3C, indole-3 carbinol; Rb, retinoblastoma protein; SVV, survivin; TGF-β, transforming growth factor-beta; ADR, adriamycin; ES2-Luc, human ovarian clear cell carcinoma cells (ES2) transfected with luciferase; A2780ADR, ADR-resistant human ovarian epithelial cancer cells.
